# Brain-derived neurotrophic factor promoter methylation and cortical thickness in recurrent major depressive disorder

**DOI:** 10.1038/srep21089

**Published:** 2016-02-15

**Authors:** Kyoung-Sae Na, Eunsoo Won, June Kang, Hun Soo Chang, Ho-Kyoung Yoon, Woo Suk Tae, Yong-Ku Kim, Min-Soo Lee, Sook-Haeng Joe, Hyun Kim, Byung-Joo Ham

**Affiliations:** 1 Department of Psychiatry, Gachon University Gil Medical Center, Incheon, Republic of Korea; 2 Department of Psychiatry, College of Medicine, Korea University, Seoul, Republic of Korea; 3 Department of Biomedical Sciences, Korea University College of Medicine, Seoul, Republic of Korea; 4 Department of Medical Bioscience, Graduate school, Soonchunhyang University, Bucheon, Republic of Korea; 5 Brain Convergence Research Center, Korea University Anam Hospital, Seoul, Republic of South Korea; 6 Department of Anatomy, College of Medicine, Korea University, Seoul, Republic of Korea

## Abstract

Recent studies have reported that methylation of the brain-derived neurotrophic factor (*BDNF*) gene promoter is associated with major depressive disorder (MDD). This study aimed to investigate the association between cortical thickness and methylation of *BDNF* promoters as well as serum BDNF levels in MDD. The participants consisted of 65 patients with recurrent MDD and 65 age- and gender-matched healthy controls. Methylation of *BDNF* promoters and cortical thickness were compared between the groups. The right medial orbitofrontal, right lingual, right lateral occipital, left lateral orbitofrontal, left pars triangularis, and left lingual cortices were thinner in patients with MDD than in healthy controls. Among the MDD group, right pericalcarine, right medical orbitofrontal, right rostral middle frontal, right postcentral, right inferior temporal, right cuneus, right precuneus, left frontal pole, left superior frontal, left superior temporal, left rostral middle frontal and left lingual cortices had inverse correlations with methylation of *BDNF* promoters. Higher levels of *BDNF* promoter methylation may be closely associated with the reduced cortical thickness among patients with MDD. Serum BDNF levels were significantly lower in MDD, and showed an inverse relationship with *BDNF* methylation only in healthy controls. Particularly the prefrontal and occipital cortices seem to indicate key regions in which *BDNF* methylation has a significant effect on structure.

A growing body of literature has suggested that brain-derived neurotrophic factor (BDNF) is closely associated with MDD. BDNF plays an important role in the survival and maintenance of cortical neurons and dendrites, as well as in synaptic plasticity^1^. Since impaired neural plasticity and neurogenesis are key mechanisms in the pathophysiology of MDD, numerous studies have reported that peripheral levels of BDNF and genetic polymorphisms of the *BDNF* gene are associated with the development and clinical course of MDD^2^,^3^.

Recent studies have increasingly focused on the possible role of *BDNF* methylation in MDD. Depression generally results from complicated interactions between genetic vulnerability and environmental stressors. With enough stress, such as early-life adversity, chromatin structure is altered without changes in the DNA sequence^4^. Methylation of CpG islands in the promoter region, which inhibits gene transcription, may be among the most common types of epigenetic mechanisms occurring in the major mental illnesses^5^.

In a rat study, *Bdnf* methylation in the hippocampus was associated with depressive-like behavior^6^. In human studies, *BDNF* methylation in DNA extracted from peripheral blood cells has been widely used as a surrogate for measuring central *BDNF* methylation levels. Several studies have reported that *BDNF* methylation measured in peripheral blood cells could be a possible diagnostic biomarker for depression^7^. Peripheral blood cell *BDNF* methylation could be altered by psychosocial stress^8^. D’Addario *et al.* suggested that patients with MDD had lower *BDNF* gene expression and higher *BDNF* methylation compared to healthy controls^9^.

If *BDNF* methylation influences the development and clinical course of MDD, the effects of this methylation may be reflected in brain structures. Methylation would inhibit expression of the BDNF gene, which in turn inhibits neurogenesis in cortex; because BDNF regulates neuronal survival, growth, immigration, axonal pruning, and dendritic growth^10^. Indeed, epigenetic controls such as DNA methylation are considered a major mechanism by which neural plasticity is altered in response to various environmental stimuli in the mature neural system^11^. Consequently, the altered neural plasticity might result in macroscopic structural changes in the cortex, which in turn result in mood dysregulation^12^.

Previously, numerous studies used voxel-based morphometry (VBM) for measuring cortical volume in MDD^13^. However, VBM measures gray matter volume, which consists of both surface area and cortical thickness. The cortical surface area and thickness have distinct cytoarchitectural and ontogenetic origins, and neurons within the cerebral cortex are organized into ontogenetic columns that run perpendicular to the surface of the brain^14^. Thus, cortical thickness reflects neural cells within a column that share a common ontogenetic origin^15^, and thereby more reliably measures changes in neural plasticity. Previously, we reported that the thickness of the prefrontal cortices were significantly decreased in patients with first-episode MDD compared to healthy controls. A study in rats suggested that *Bdnf* methylation resulted in decreased *Bdnf* mRNA expression in the prefrontal region^16^. However, despite the critical role of BDNF in determining cortical thickness, to the best of our knowledge, no studies have investigated the relationship between *BDNF* methylation and cortical thickness in patients with MDD.

In this study, we investigated differences in *BDNF* promoter methylation and cortical thickness between patients with recurrent MDD and healthy controls. We hypothesized that patients with MDD would have higher *BDNF* promoter methylation as well as thinner cortices than healthy controls. Since abnormal epigenetic regulation of the *BDNF* gene would result in neurodegenerative changes and consequently decreased cortical thickness in patients with MDD, the relationship between the thinner cortices and *BDNF* promoter methylation in patients with MDD would likely have an inverse correlation. Additionally, since increased *BDNF* methylation might reduce bioavailability of serum BDNF levels, there is a possibility that *BDNF* methylation and serum BDNF levels are inversely correlated. However, to the best of our knowledge, only a recent study with only 11 patients with MDD reported a possible association between serum BDNF levels and *BDNF* methylations^17^. This study reported serum BDNF levels and *BDNF* methylation to be inversely correlated with total methylation rates, whereas there was no association with each CpG site. Thus, we measured serum BDNF levels together with *BDNF* methylation to investigate more comprehensively, the correlations between each factors.

On the other hand, recent studies have reported that first-episode patients with MDD might have increased volume in the cortical^18^ and hippocampal^19^ regions compared to healthy controls. Initial inflammatory processes are thought to play a neuroprotective function and may temporarily increase cortical volumes^20^, although the exact mechanisms should be further studied. Therefore, to reliably examine the possible effects of *BDNF* promoter methylation on neurodegenerative changes as represented by cortical thickness, we recruited only patients with recurrent MDD.

## Results

### Sociodemographic, clinical, and genetic data

A total of 130 subjects were recruited. They consisted of 65 patients with recurrent MDD and 65 healthy controls. Sociodemographic and clinical data for the subjects are presented in Table 1. There were no significant differences in age and gender between the two groups. Patients with recurrent MDD had significantly fewer years of education, less employment, and a higher family history of MDD compared to healthy controls. Sixteen out of 65 (24.6%) patients with MDD had more than three previous depressive episodes. Genotyping of *BDNF* for one healthy control failed. The distribution of polymorphisms in rs6265 was fit to HWE for each group.

Patients with MDD had significantly higher rates of methylation at CpG2 and CpG4 than healthy controls. There were no significant differences in total intracranial volume (TIV) between patients with recurrent MDD and healthy controls. There were no association between duration of illness and *BDNF* promoter methylation at CpG1 (*r* = −0.190, *p* = 0.136), CpG2 (*r* = 0.020, *p* = 0.879), CpG3 (*r* = −0.132, *p* = 0.302), and CpG4 (*r* = 0.019, *p* = 0.884).

With regard to the comparison of BDNF promoter methylation between medication-naïve and on-medication patients with MDD, there were no differences (Supplementary Table 1).

In the 33 patients with MDD who took antidepressants, more than half of patients (17 out of 33, 51.5%) took antipsychotics. The most frequently prescribed antidepressants and atypical antipsychotics were escitalopram and quetiapine, respectively.

### Comparisons of cortical thickness between patients with MDD and healthy controls

Patients with MDD had significantly thinner right medial orbitofrontal, right lingual, right lateral occipital, left lateral orbitofrontal, left pars triangularis, and left lingual cortices as compared to healthy controls (Table 2).

### Correlations between severity of depression and cortical thickness

The 17-item Hamilton Rating Scale for Depression (HRSD) scores, which represent severity of depression showed positive correlation with left supramarginal cortex and negative correlation with left lingual cortex among MDD group (Supplementary Table 2).

### Correlations between *BDNF* promoter methylation and cortical thickness

*BDNF* promoter methylation at CpG2 and CpG4, which were higher in patients with MDD than healthy controls, were used for the correlation analysis.

**Figure 1:**
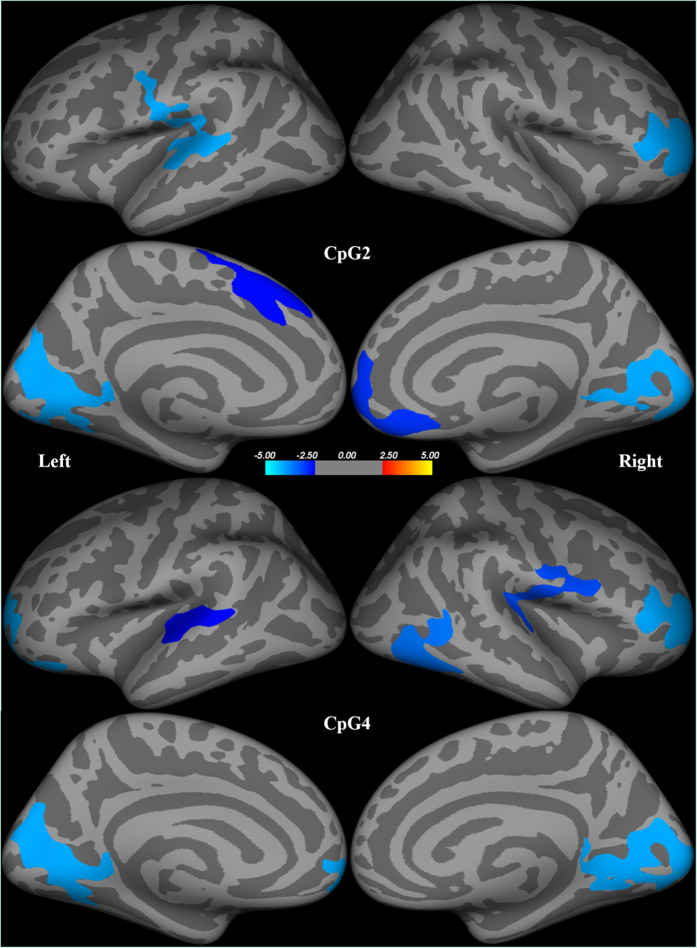
Correlations between brain-derived neurotrophic factor (*BDNF*) methylation and cortical thickness among patients with major depressive disorder. The inflated maps illustrate brain regions with Monte-Carlo permutation test-adjusted family-wise error (*p* < 0.05). The top four brain maps show correlations between *BDNF* methylation at CpG2 and cortical thickness in the left and right hemisphere, respectively. The bottom four brain maps show correlations between *BDNF* methylation at CpG4 and cortical thickness in the left and right hemisphere, respectively. The colored areas represent maximum *z* scores in each cluster.

**Figure 2:**
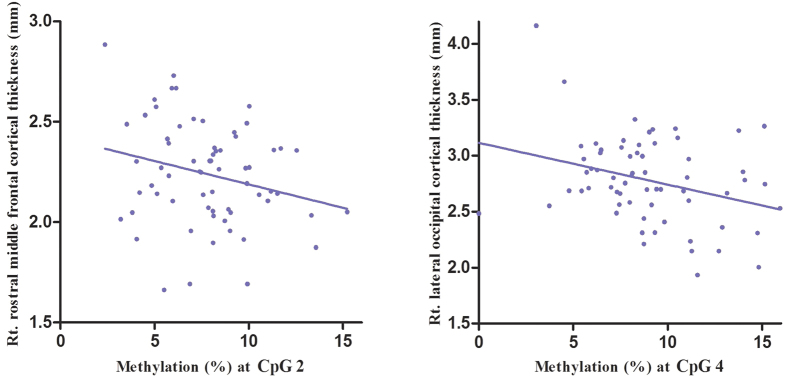
Scatter plots of correlations between brain-derived neurotrophic factor (*BDNF*) methylation and the estimated cortical thickness among patients with major depressive disorder.

Among MDD group, *BDNF* promoter methylation at CpG2 had inverse correlations with thickness in right pericalcarine, right inferior temporal, right medical orbitofrontal, right rostral middle frontal, left superior frontal, left superior temporal, and left lingual, and right rostral middle frontal cortices. *BDNF* promoter methylation at CpG4 had also inverse correlations with thickness in right rostral middle frontal, right medial orbitofrontal, right cuneus, right precuneus, right postcentral, left lingual, left superior frontal, left superior temporal, and left frontal pole cortices (Table 3, Figs 1 and 2). There were no cortical regions having positive correlations with *BDNF* promoter methylation.

As noted in the Table 3, several of the cortical regions thinned in patients with MDD were also inversely correlated with *BDNF* promoter methylation. Right medial orbitofrontal and left lingual cortices were inversely correlated with *BDNF* promoter methylation both at CpG2 and CpG4.

Among medicated MDD group, *BDNF* promoter methylation at CpG4 had inverse correlations with thickness in right inferior temporal, right pericalcarine, left rostral middle frontal, and left lingual cortices (Supplementary Table 4).

There were no correlations between *BDNF* promoter methylation at any CpG sites and cortical thickness among healthy controls (Supplementary Figures 1 and 2).

There were no *BDNF* promoter methylation and genotypes on cortical thickness at any CpG sites among MDD and healthy controls, respectively.

### Correlations between serum BDNF levels and cortical thickness

There were no association between serum BDNF levels and cortical thickness both in MDD and healthy controls, respectively.

### Correlations between *BDNF* promoter methylation and depressive symptoms

There were no correlations between BDNF promoter methylation and HRSD scores both in the healthy controls and MDD, respectively (Supplementary Table 5).

### Correlations between *BDNF* promoter methylation and serum BDNF levels

Among healthy controls, *BDNF* promoter methylation at CpG2 (r = −0.264, p = 0.038) and CpG3 (r = −0.433, p < 0.001) had inverse correlations with serum BDNF levels (Supplementary Table 6). There were no association between *BDNF* promoter methylation and serum BDNF levels at CpG1 and CpG4.

There were no association between *BDNF* promoter methylation and serum BDNF levels at any CpG sites among patients with MDD.

## Discussion

In this study, we investigated the association between *BDNF* promoter methylation and cortical thickness among patients with recurrent MDD. Patients with recurrent MDD had thinner right medial orbitofrontal, right rostral middle frontal, right superior temporal, right middle temporal, and right lingual cortices. In the left hemisphere, the MDD group had thinner lateral orbitofrontal, pars triangularis, precuneus, lingual, and lateral occipital cortices. There was no cortical region in which healthy controls had thinner cortex compared to the patients with recurrent MDD. Regarding *BDNF* promoter methylation, as expected, patients with MDD had higher methylation levels than healthy controls.

As briefly mentioned in the Introduction section, peripheral *BDNF* promoter has been increasingly used in the field of epigenetics of major psychiatric disorders. Recent studies reported that peripheral molecular BDNF, such as methylation, closely reflect central activity of the BDNF in brain regions closely related with mood regulation. In a postmortem study conducted on bipolar patients, *BDNF* promoter methylations in the peripheral tissues and quadriceps tissues, were positively associated with those in the hippocampus of patients with bipolar disorder^21^. The authors of this study argued that the results supported the usefulness of peripheral *BDNF* methylation as a surrogate for central *BDNF* methylation. In a rat study, *Bdnf* methylation resulted in decreased *Bdnf* mRNA expression in the prefrontal region^16^. The results suggest that peripheral *BDNF* promoter methylation could reflect molecular abnormalities in the CNS, particularly in areas where a lot of BDNF receptors are present, such as the hippocampus and prefrontal cortex. There have been increasing evidences more directly supporting the association between peripheral *BDNF* methylation and depression. Both *BDNF* methylation and gene expression of peripheral blood mononuclear cells were higher in patients MDD as compared to healthy controls^9^. Additionally, higher *BDNF* methylation exerted negative influence on the depressive severity during 1-year follow up period^22^.

One of the most interesting findings is that *BDNF* promoter methylation and cortical thickness had inverse correlation among patients with MDD, but not healthy controls. In particular, as described in the Results section, prefrontal and occipital cortices which were found to be thinner in patients with MDD also had inverse correlations with *BDNF* promoter methylation. Additionally, the prefrontal and occipital cortices had more frequent and significant associations with *BDNF* promoter methylation than other cortical areas. Right medial orbitofrontal and left lingual cortices were thinned in patients with MDD and also had inverse correlations with *BDNF* promoter methylation at CpG2 or CpG4, which were significantly higher in patients with MDD as compared to healthy controls.

Our results could be interpreted in several perspectives. First, there is a possibility that abnormalities in the frontal and occipital cortices might occur together, perhaps coupled with a neural pathway. In studies using diffuse tensor imaging (DTI), fractional anisotropy (FA) in the inferior fronto-occipital fasciculus was decreased in subpopulations of MDD such as early-life adversity^23^. A meta-analysis also reported that the inferior fronto-occipital fasciculus was one of the main tracts involved in MDD^24^. As BDNF is a neurotrophic factor involving neuronal survival, migration, growth, synaptogenesis, and neuroplasticity^25^,^26^, it is plausible to suggest that genetics of BDNF, such as polymorphisms and methylation, might have influence on the disruption of the fronto-occipital white matter tract. A DTI study revealed that healthy adults with *BDNF* val/val polymorphisms had decreased FA in the prefrontal and occipital pathway^27^.

Second, decreased cortical thickness and inverse relationship with *BDNF* methylation of the prefontal and occipital regions, could be considered as an individual anatomical region. The prefrontal cortex is one of the most widely investigated regions for major psychiatric disorders including MDD. The prefrontal cortex regulates emotion and executive functions, and numerous studies have suggested that prefrontal dysfunction is associated with depressive mood, lack of motivation, and executive dysfunction, all of which belong to the essential features of MDD^28^,^29^,^30^. Hence, many neuroimaging studies on MDD have focused on, or reported results regarding the prefrontal cortex^31^. The possible role of dysfunctional BDNF activity in the prefrontal cortex in depression may be mediated by the BDNF-neurotrophin receptor tyrosine kinase 2 signaling pathway, which contributes to the molecular vulnerability in depression^32^.

However, the association between the occipital cortex and MDD has recently received much attention. A previous study has suggested a thinner occipital cortex to be an endophenotype for MDD^33^. Subsequent studies have reported that abnormalities in the occipital lobe might be closely associated with recurrent and treatment-refractory depression^34^,^35^. We have revealed in our previous study, that lingual gyrus volume was associated with treatment response in MDD^34^. In this study, patients who were non-responsive to antidepressants had a smaller gray matter volume in the lingual gyrus as compared to antidepressant-responsive patients with MDD. Our results are also in accordance with major findings of recent studies. Maller *et al.* reported that occipital bending, which refers to a phenomenon in which the occipital lobe wraps around other brain regions, is found more frequently in treatment-refractory MDD patients than in healthy controls^35^. Whereas the bending of this brain region was viewed primarily from the perspective of brain asymmetry, the authors suggested that underlying neurotrophic mechanisms such as altered neuronal pruning should be considered.

We speculate that BDNF may play an important role in the association between the occipital cortex and MDD. Besides its neurotrophic role, BDNF also enhances formation and maintenance of excitatory glutamatergic and inhibitory GABAergic neuronal synapses^36^. When BDNF activity is disturbed, the balance between inhibitory and excitatory neurotransmission is upset. Inhibitory GABAergic neurons are primarily modulated by BDNF in particular, as BDNF acts differentially on GABAergic and glutamatergic neurons^37^. Whereas transcellular transfer of BDNF is crucial in the development and maturation of GABAergic neurons, it is not so correlated with dendritic development of glutamtergic neurons^38^,^39^. A recent mice study also suggested that activity-driven *Bdnf* influenced only GABAergic neurons, but not glutamatergic neurons. Thus, *BDNF* methylation might result in decreased GABAergic neurotransmission. Many studies have reported that the occipital cortex has higher GABA concentration compared to other brain regions^38^. Occipital GABA concentrations are reportedly decreased in patients with MDD compared to healthy controls^39^. The same study group also suggested that occipital GABA might be a biological marker for treatment response in MDD^40^. In that study, glutamate levels were decreased in the occipital cortex, suggesting that abnormal inhibitory-excitatory neurotransmission might be a neural substrate for MDD. Given the results of previous studies along with our study, we speculate that *BDNF* methylation might substantially damage the occipital cortex, in which GABA concentrations are the heist in the brain. However, it remains to be revealed how *BDNF* methylation-associated occipital cortical thinning contributes to the symptoms of depression.

It is of interest that *BDNF* promoter methylation showed negative correlations with serum BDNF levels only in healthy controls, but not in MDD patients. Since methylation inhibits expression of the *BDNF* gene, the negative correlations between methylation and serum levels of BDNF seem to be expectable. The significantly higher *BDNF* promoter methylation together with the dissociation between methylation and serum BDNF levels among MDD patients, might be interpreted in perspective of the loss of physiological or normal regulatory process of BDNF, which is presented in healthy controls.

The relationship between cortical thickness and severity of depression remains inconclusive. In this study, the HRSD, which represents severity of depression, showed negative correlations with left lingual cortex thickness, but positive correlations with left supramarginal cortex thickness. The negative correlation between HRSD and left lingual cortex thickness may be viewed as a representation of the association between the occipital cortex and depression, as described above. On the other hand, the positive correlation between supramarginal cortical thickness and HRSD scores suggests that one should be cautious when making the assumption that reductions in thickness will equate to reductions in depressive symptoms. Whereas a recent study reported that left supramarginal cortical thickness was increased in drug-naïve, first-episode MDD patients as compared to healthy controls^41^, there was no difference between MDD patients and healthy controls in our study. This raises the question on whether the positive correlation between left supramarginal cortical thickness and HRSD scores had clinical impact on MDD patients. Previous studies on brain volume and depressive symptom severity have yielded conflicting results. Among them, most studies found no correlations between depressive symptom severity and brain volume^42^,^43^,^44^, but some studies found positive^45^,^46^ as well as negative correlations^47^ with subcortical brain volume. Recent meta-analyses showed no evidence of an association between symptom severity and subcortical gray matter volumes, and it was suggested that assessing the severity of depression with HDRS sores at study inclusion could not fully characterize the severity of the entire depressive episode^48^. Future research could further investigate associations between cortical thickness and depression severity using a larger cohort of patients, and prospective longitudinal designs.

Lastly, there were no association between serum BDNF levels and cortical thickness among patients with MDD and healthy controls, respectively. Recent two studies reported that serum BDNF levels were differently associated with cortical thickness in schizophrenia as compared to healthy controls^49^,^50^. However, to the best of our knowledge, no studies investigated relationships between serum BDNF levels and cortical thickness in MDD, whereas one study revealed association between *BDNF* polymorphism and cortical thickness at *a priori* regions of interests (amygdala, anterior cingulate, middle frontal cortex, and orbitofrontal cortex)^51^. Thus, further studies are needed for comprehensive investigation for association between various BDNF measures (methylation, genotype, and peripheral levels) and cortical thickness in MDD.

Our study had several limitations. First, approximately half of the patients with recurrent MDD had taken antidepressants before the study. Similar to previous studies that included patients taking antidepressants^52^, *BDNF* polymorphism allele type was not significantly different between patients with MDD and healthy controls. There is a possibility that antidepressants might influence methylation^53^. Thus, the results seen in patients with recurrent MDD should be carefully interpreted. Second, this study used a cross-sectional design and not longitudinal. Thus, although we statistically demonstrated there was no association between duration of illness and *BDNF* methylation at CpG sites, we could not clearly confirm how early these changes in *BDNF* methylation and cortical thickness among patients with MDD appear. The cross-sectional design of our study may also act as a limitation in comprehensively elaborating the complex associations among *BDNF* methylation, serum BDNF levels, HRSD scores, and cortical thickness. Whereas *BDNF* methylation and cortical thickness consistently showed inverse correlations, serum BDNF levels and HRSD scores did not have significant relationships with *BDNF* methylation and/or cortical thickness. Serum BDNF levels are state-dependent, which are decreased predominantly during depressive periods^54^. Although more studies are needed to investigate whether the *BDNF* methylation is state- or trait –dependent, several studies have supported that *BDNF* methylation has no association with the severity of the current depressive episode^9^,^22^,^55^. One study revealed that *BDNF* methylation had no association with baseline severity of depression, but had an inverse relationship with 1-year follow up severity of depression^22^, raising a possibility that *BDNF* methylation would influence the longitudinal prognosis of depressive disorder, rather than the current state of depression. Third, we analyzed methylation at only four CpG sites, which is too few to determine the overall effects of these methylations. Third, we did not measure early life adversity, which has been reported to be associated with methylation of *BDNF* promoters among healthy controls and patients with MDD. However, the focus of our study was not to examine the lasting epigenetic effects of early life adversity, which have already been well investigated^16^. Rather, we aimed to investigate whether the differential levels of *BDNF* methylation between patients with MDD and healthy controls had any correlation with the cortical thickness of various brain regions. Lastly, since the types of antidepressants, mood stabilizers, and atypical antipsychotics were diverse, we could not control for the individual effects of antidepressants.

In summary, our study provides the first evidence of an association between *BDNF* promoter methylation and cortical thickness in patients with recurrent MDD. Cortical thinning, particularly in the prefrontal and occipital cortices, seems to indicate key regions in which *BDNF* methylation has a significant effect on structure. Further studies should elucidate the neuromolecular mechanisms underlying these interactions as well as their clinical outcomes.

## Methods

### Participants and procedures

All subjects were aged 18 to 65 and were recruited at the Korea University Anam Hospital. All patients with recurrent MDD were required have full interepisode recovery not being met depressed episode. Some of subjects in this study were included in previous studies^56^. The Edinburgh Handedness Test^57^ was applied to all participants before imaging, and only those who were right-handed were included in this study. Among all participants, an Axis I diagnosis was determined by a board-certified psychiatrist according to the Diagnostic and Statistical Manual for Mental Disorders, fourth edition (DSM-IV)^58^, using the Korean version of the Structured Clinical Interview for DSM-IV Axis-I Disorders^59^. We contacted their close relatives in order to gather available data on social, demographic, lifestyle, personality and clinical variables, for a more reliable diagnosis of a past major depressive episode. Finally, we formulated a lifetime mood chart which we used to diagnose recurrent depression. Exclusion criteria were as follows: (1) a past history or current diagnosis of comorbid axis I or II disorders according to DSM-IV criteria, (2) an IQ score under 80, (3) a history of primary neurologic diseases, such as Parkinson’s disease or epilepsy, (4) intracranial lesions such as space-occupying lesions or cerebrovascular diseases, or (5) any contraindications for magnetic resonance imaging (MRI) such as pacemakers. Age- and gender-matched healthy controls were recruited. The healthy controls were confirmed to have no present or past history of psychiatric illnesses by board-certified psychiatrists. Depression was measured by the HRSD. At the time of enrollment, 33 out of 65 patients were on antidepressant treatment with flexible dose, whereas 32 patients were medication-naïve.

All participants gave written informed consent after a full explanation and understanding of this study. The study protocol was approved by the Institutional Review Board of Korea University Anam Hospital and was conducted in accordance with the Declaration of Helsinki.

### Selection of genomic regions of the *BDNF* gene for methylation analysis

We used whole blood to conduct genetic analyses. We measured methylations at four CpG sites (CpG1 = −675, CpG2 = −682, CpG3 = −686, and CpG4 = −688, distance [nt] from transcription start site [+1]). The above CpG regions were selected based on a study showing that methylation of analogous regions of rat *Bdnf* led to decreased *BDNF* mRNA expression in the prefrontal cortex^16^. A previous study also examined those regions in humans^60^.

### *BDNF* gene methylation analysis

Detailed methods for the BDNF gene methylation analysis were described in our previous study^56^.

### MRI acquisition

Three-dimensional structural MRI scans were acquired with a 3.0 T Siemens Trio whole-body imaging system (Siemens Medical Systems, Iselin, NJ, USA), using a T1-weighted magnetization-prepared rapid gradient-echo (MP-RAGE [1900 ms repetition time, 2.6 ms echo time, 220 mm field of view, 256 × 256 matrix size, 176 coronal slices without gap, 1 × 1 × 1 mm, 3 voxels, 16° flip angle, number of excitations=1]).

### MRI processing for cortical thickness

Cortical thickness was defined as the shortest distance between gray/white matter boundary and the pial surface at each point across the cortical mantle. Cortical thickness was automatically estimated using FreeSurfer (software version 5.0, http://surfer.nmr.mgh.harvard.edu). The technical details of measuring cortical thickness and TIV using FreeSurfer have been described elsewhere^61^. We carefully inspected all raw images at segmented and inflated stages, and we confirmed that no images had substantial defects. For further analysis, cortical maps were smoothed using a Gaussian kernel with a full width at half maximum of 10 mm.

### Statistical analysis

Sociodemographic and clinical data were compared between patients with MDD and healthy controls with a chi-square test for dichotomous variables, and an independent *t*-test for continuous variables. *BDNF* promoter methylation at CpG sites where there were significant differences between MDD and healthy controls were used in the subsequent analysis for correlations between methylation and cortical thickness. Partial correlation analysis adjusting age and gender was conducted to identify association between duration of illness and *BDNF* promoter methylation at CpG sites. The statistical analysis was conducted using SPSS version 12.0 (SPSS Inc., Chicago, IL, US).

In the analyses of cortical thickness, a vertex-wise general linear model was used for detecting the main effects of diagnosis for cortical thickness between patients and controls. Correlations between *BDNF* promoter methylation and the estimated cortical thickness among MDD and healthy controls were analyzed, respectively. Based on a previous study^62^ and heterogeneous clinical status of patients, age, gender, intracranial volume, and severity of depression were included as covariates. Several additional analyses were also conducted to identify possible role of genetic or clinical variables. To control for possible effects of polymorphisms of *BDNF* genotypes, correlations between *BDNF* promoter methylation and cortical thickness according to genotypes (AA vs. GG) were analyzed in MDD and health controls, respectively. To control for possible effects of being treated with medication, correlations between *BDNF* promoter methylation and cortical thickness additionally adjusted for the presence of medication were analyzed among MDD. Correlations between clinical variables, including HRSD scores and period of medications, and cortical thickness were also analyzed. Lastly, an analysis was conducted to examine possible associations between serum BDNF levels and *BDNF* methylations as well as cortical thickness.

To prevent a type I error from multiple comparisons, the Monte-Carlo permutation test implemented in FreeSurfer as applied, and the statistical significance level was considered at cluster-wise probability (CWP), which is similar to alpha significance, *p* < 0.05.

## Additional Information

**How to cite this article**: Na, K.-S. *et al.* Brain-derived neurotrophic factor promoter methylation and cortical thickness in recurrent major depressive disorder. *Sci. Rep.* **6**, 21089; doi: 10.1038/srep21089 (2016).

## Supplementary Material

Supplementary Information

## Figures and Tables

**Table 1:** Demographic and clinical data for patients with recurrent major depressive disorder and healthy controls.

	MDD (n = 65)	HC (n = 65)	*t* or *χ*^*2*^
Age	42.52 (11.42)	40.34 (13.94)	−0.977
Sex, female	54	50	0.769
Education, <12 years	13 (20.0)	8 (12.3)	1.420
Married, yes	39 (60.0)	43 (66.2)	0.528
Occupation, yes	43 (66.2)	44 (67.7)	0.035
Family history of MDD, yes^*^	20 (30.8)	1 (1.5)	20.502
HRSD scores^*^	16.00 (8.2)	2.03 (2.24)	−13.23
Duration of illness (months)	47.60 (50.0)		
Medication			
Naïve	32		
On-medication	33		
Type of regimen			
Monotherapy	18		
Antidepressants only	17		
Mood stabilizer only	1		
Polypharmacy	15		
Antidepressants + antidepressants	7		
Antidepressants + atypical antipsychotics	6		
Antidepressants + atypical antipsychotics + mood stabilizers	2		
Duration of medication (months)	38.36 (65.1)		
Past history of Suicide attempt	8 (12.3)		
Genotype of *BDNF*			1.687
AA	15 (23.1)	13 (20.3)	
AG	38 (58.5)	33 (51.6)	
GG	12 (18.5)	18 (28.1)	
*BDNF* Methylation (%)			
CpG1	7.96 (2.79)	7.91 (3.06)	−0.098
CpG2^*^	7.84 (2.69)	6.34 (3.09)	−2.942
CpG3	6.24 (2.55)	6.99 (3.29)	1.465
CpG4^*^	8.96 (3.20)	7.18 (3.50)	−3.025
Serum BDNF levels (ng/ml) ^*^	3.73 (3.93)	7.55 (5.33)	4.518
Total intracranial volume (cm^3^)	1278.93 (142.69)	1287.13 (147.79)	0.322

All data are represented as mean (*SD*) or number (%).

MDD: major depressive disorder, HC: healthy controls, HRSD: Hamilton Rating Scale for Depression, BDNF: Brain-derived neurotrophic factor

^*^p < 0.05.

**Table 2:** Differences in cortical thickness between patients with major depressive disorder and healthy controls.

Cortical area	Maximum	Cluster size (mm^2^)	Talx	Taly	Talz	CWP
Right hemisphere						
Medial orbitofrontal	−4.836	3506.31	5.9	28.3	−21.8	0.0001
Lateral occipital	−2.867	1383.22	40.3	−68.4	1.4	0. 001
Lingual	−2.880	1679.78	11.2	−81.3	−2.5	0.0001
Left hemisphere						
Lingual	−5.494	1298.90	−4.8	−80.6	0.7	0.0018
Pars triangularis	−4.240	1021.73	−46.6	29.1	7.7	0.0111
Lateral orbitofrontal	−3.190	2888.04	−22.7	39.7	−12.2	0.0001

Cortical regions analyzed by Monte-Carlo permutation test-adjusted family-wise error (*p* < 0.05) are presented. CWP: cluster-wise p value.

**Table 3:** Correlations between brain-derived neurotrophic factor methylation and cortical thickness among major depressive disorder.

Cortical area	Maximum	Cluster size (mm^2^)	Talx	Taly	Talz	CWP
CpG2						
Right rostral middle frontal	−4.144	2012.51	26.4	47.2	−2.0	0.0001
Right inferior temporal	−4.486	1036.76	44.7	−56.3	−5.2	0.0130
Right medial orbitofrontal^*^	−4.306	1289.51	8.2	58.5	−4.9	0.0025
Right pericalcarine	−3.884	2543.57	13.4	−87.1	10.8	0.0001
Left lingual^*^	−7.245	3200.38	−7.0	−65.2	6.2	0.0001
Left superior frontal	−4.489	1071.30	−10.8	16.6	40.7	0.0081
Left superior temporal	−3.373	1843.05	−53.8	−28.3	5.9	0.0001
Left rostral middle frontal	−3.340	939.09	−35.9	41.2	8.4	0.0193
CpG4						
Right rostral middle frontal	−3.714	1819.02	27.7	47.1	−2.3	0.0001
Right cuneus	−5.447	3730.98	4.4	−71.9	19.8	0.0001
Right medial orbitofrontal^*^	−4.639	855.23	8.2	58.5	−4.9	0.0450
Right precuneus	−4.872	850.31	6.4	−41.5	40.3	0.0466
Right postcentral	−4.263	1348.42	57.5	−10.2	13.1	0.0013
Left lingual^*^	−7.476	2905.29	−7.1	−64.2	5.6	0.0001
Left superior temporal	−4.259	1062.24	−53.9	−20.3	1.4	0.0085
Left superior frontal	−3.390	851.00	−10.5	17.4	42.2	0.0364
Left frontal pole	−2.995	1674.28	−11.3	54.6	−14.2	0.0001

Cortical regions analyzed by Monte-Carlo permutation test-adjusted family-wise error (*p* < 0.05) are presented. MDD: major depressive disorder. CWP: cluster-wise *p* value.

^*^Cortical regions thinner in patients with MDD as compared to healthy controls.
