# Supplementary material for: Brain-derived neurotrophic factor promoter methylation and cortical thickness in recurrent major depressive disorder
Source: Sci Rep. 2016 Feb 15;6:21089. doi: 10.1038/srep21089 (PMC4753411; doi:10.1038/srep21089)
Supplement: Supplementary Information [file srep21089-s1.doc]

**Brain-derived neurotrophic factor promoter methylation and cortical thickness in recurrent major depressive disorder**

Kyoung-Sae Na^1^, Eunsoo Won^2^, June Kang^3^, Hun Soo Chang^4^, Ho-Kyoung Yoon^2^, Woo Suk Tae^5^, Yong-Ku Kim^2^, Min-Soo Lee^2^, Sook-Haeng Joe^2^, Hyun Kim^6†^, Byung-Joo Ham^2†^

^1^Department of Psychiatry, Gachon University Gil Medical Center, Incheon, Republic of Korea

^2^Department of Psychiatry, College of Medicine, Korea University, Seoul, Republic of Korea

^3^Department of Biomedical Sciences, Korea University College of Medicine, Seoul, Republic of Korea

^4^Department of Medical Bioscience, Graduate school, Soonchunhyang University, Bucheon

^5^Brain Convergence Research Center, Korea University Anam Hospital, Seoul, South Korea

^6^Department of Anatomy, College of Medicine, Korea University, Seoul, Republic of Korea

^†^Corresponding Authors:

[hambj@korea.](mailto:hambj@korea.)ac.kr

[kimhyun@korea.ac.kr](mailto:kimhyun@korea.ac.kr)

**Supplementary Table 1. Comparison of brain-derived neurotrophic factor promoter methylation between medication-naïve and on-medication patients with MDD**

|  | **Drug-naïve** | **On-medication** | ***t* or *χ^2^*** |
| --- | --- | --- | --- |
| **CpG 1** | 8.18 (2.61) | 7.75 (2.98) | 0.627 |
| **CpG 2** | 7.44 (2.56) | 8.22 (2.80) | -1.177 |
| **CpG 3** | 6.00 (2.47) | 6.46 (2.65) | -0.731 |
| **CpG 4** | 8.20 (3.32) | 9.69 (2.94) | -1.917 |

All data are represented as mean (*SD*).

**Supplementary Table 2. Correlations between severity of depression and cortical thickness among** major depressive disorder

| **Cortical area** | **Maximum** | **Cluster size (mm^2^)** | **Talx** | **Taly** | **Talz** | **CWP** |
| --- | --- | --- | --- | --- | --- | --- |
| **Left lingual** | -2.833 | 1342.04 | -13.3 | -76.9 | -1.5 | 0.0013 |
| **Left supramarginal** | 3.144 | 1050.99 | -61.9 | -27.6 | 24.7 | 0.0093 |

| **Cortical area** | **Maximum** | **Cluster size (mm^2^)** | **Talx** | **Taly** | **Talz** | **CWP** |
| --- | --- | --- | --- | --- | --- | --- |
| **CpG2** |  |  |  |  |  |  |
| **Right rostral middle frontal** | -4.872 | 2020.05 | 27.5 | 47.1 | -0.8 | 0.0001 |
| **Left lingual**^*^ | -5.856 | 2459.62 | -9.3 | -65.1 | 6.0 | 0.0001 |
| **CpG4** |  |  |  |  |  |  |
| **Right cuneus** | -5.447 | 3730.98 | 4.4 | -71.9 | 19.8 | 0.0001 |
| **Right lingual**^*^ | -4.408 | 1392.60 | 21.2 | -51.7 | 0.2 | 0.0010 |
| **Right pars triangularis** | -4.222 | 1084.69 | 44.6 | 34.8 | 1.5 | 0.0094 |
| **Left lingual**^*^ | -9.199 | 1594.95 | -9.3 | -65.1 | 6.0 | 0.0001 |
| **Left pars orbitalis** | -4.065 | 990.93 | -45.7 | 32.4 | -10.6 | 0.0136 |

**Supplementary Table 3. Correlations between brain-derived neurotrophic factor promoter methylation and cortical thickness after adjusting for being treated with medication among major depressive disorder**

**Supplementary Table 4. Correlations between brain-derived neurotrophic factor methylation and cortical thickness among major depressive disorder (MDD) after adjusting for duration of medication among on-medication MDD**

| **Cortical area** | **Maximum** | **Cluster size (mm^2^)** | **Talx** | **Taly** | **Talz** | **CWP** |
| --- | --- | --- | --- | --- | --- | --- |
| **CpG4** |  |  |  |  |  |  |
| **Right inferior temporal** | -5.335 | 1731.91 | 47.6 | -53.8 | -8.1 | 0.0001 |
| **Right pericalcarine** | -4.083 | 1765.79 | 16.6 | -89.3 | 5.8 | 0.0001 |
| **Left Lingual**^*^ | -4.693 | 3931.63 | -11.5 | -55.0 | 1.5 | 0.0001 |
| **Left rostral middle frontal** | -2.345 | 808.07 | -32.0 | 49.1 | 1.4 | 0.0496 |

**Supplementary Table 5. Correlations between severity of depression and brain-derived neurotrophic factor promoter methylation.**

|  | **Major depressive disorder** | **Healthy controls** |
| --- | --- | --- |
| **CpG 1** | 0.074 (0.560) | 0.077 (0.543) |
| **CpG 2** | -0.030 (0.810) | 0.034 (0.787) |
| **CpG 3** | 0.041 (0.745) | -0.037 (0.771) |
| **CpG 4** | -0.029 (0.817) | 0.001 (0.992) |

All data are represented as coefficient *r* (*p* values)

**Supplementary Table 6. Correlations between brain-derived neu**rotrophic factor (BDNF) promoter methylation and serum BDNF levels

|  | **Major depressive disorder** | **Healthy controls** |
| --- | --- | --- |
| **CpG 1** | 0.111 (0.394) | 0.037 (0.777) |
| **CpG 2** | -0.264 (0.968) | -0.264 (0.038) |
| **CpG 3** | -0.048 (0.715) | -0.433 (0.000) |
| **CpG 4** | -0.005 (0.972) | -0.030 (0.814) |

All data are represented as coefficient *r* (*p* values)

**Supplementary Figure 1. Correlations between brain-derived neurotrophic factor (*BDNF*) methylation and cortical thickness at CpG 1 and CpG 2 among healthy controls. The inflated maps illustrate brain regions with uncorrected *p* values (*p* < 0.05).**

**
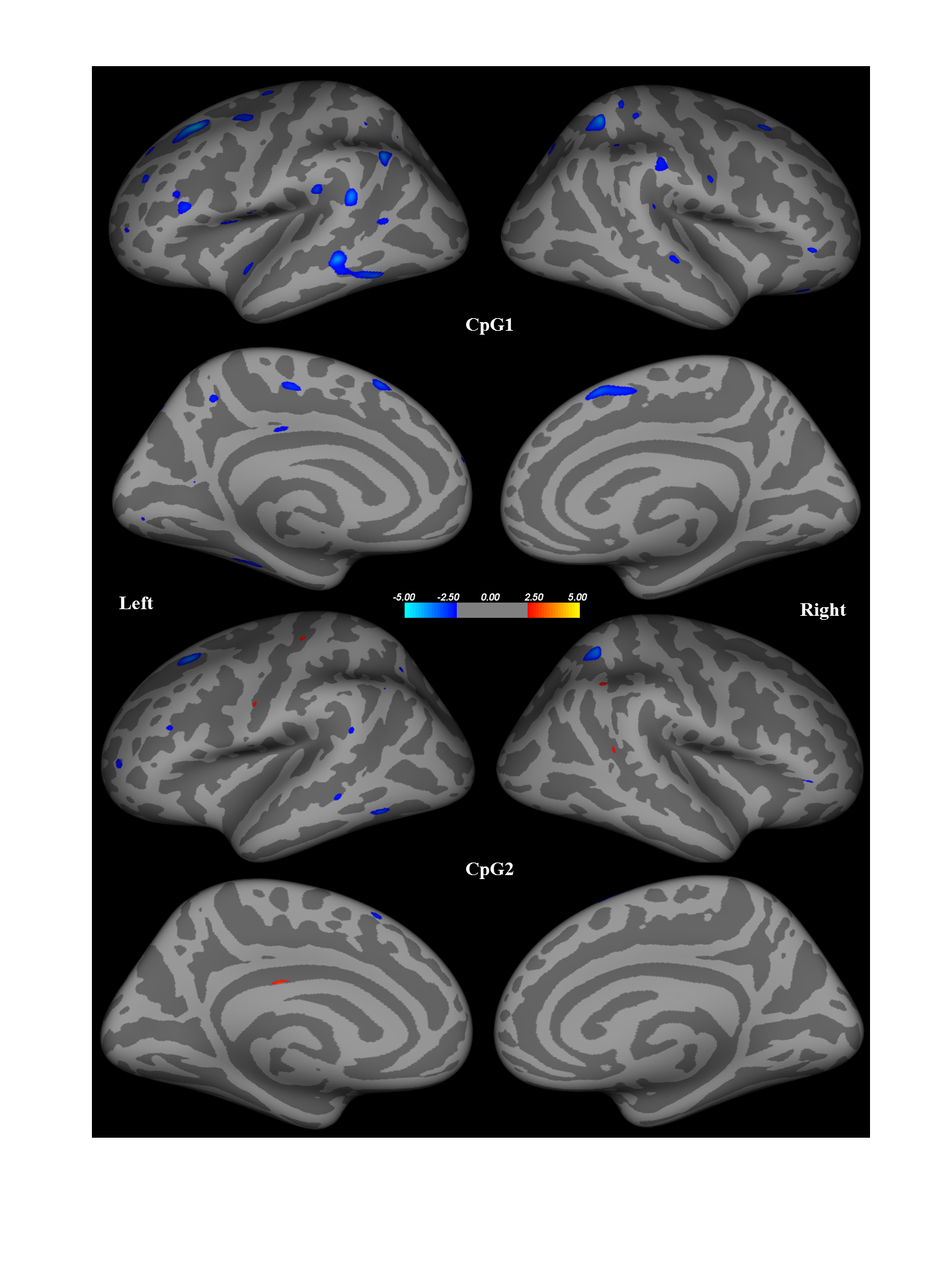
**

**Supplementary Figure 2. Correlations between brain-derived neurotrophic factor (*BDNF*) methylation and cortical thickness at CpG 3 and CpG 4 among healthy controls. The inflated maps illustrate brain regions with uncorrected *p* values (*p* < 0.05).**

**
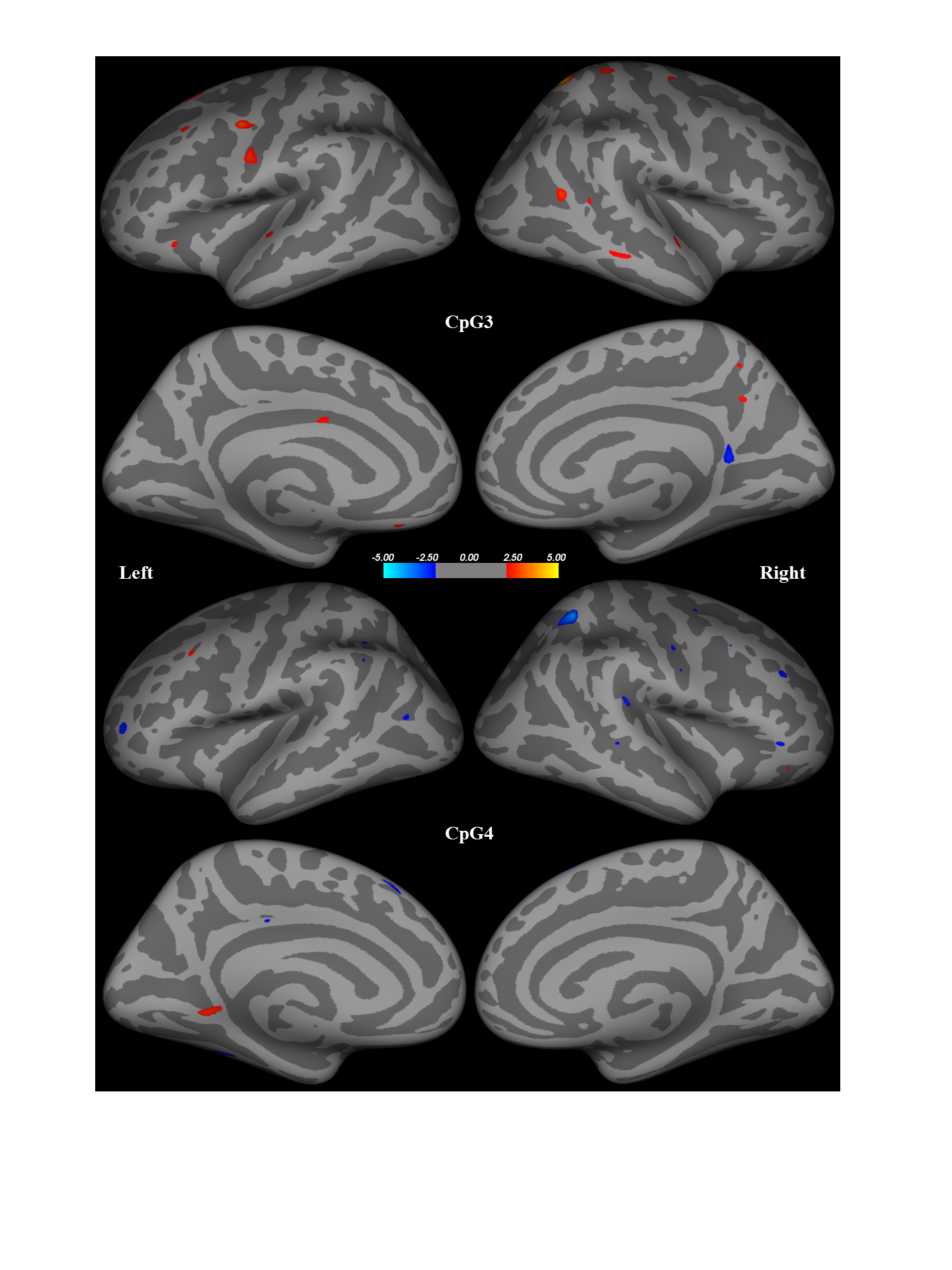
**
